# Supplementary material for: Stroke but no hospital admission: Lost opportunity for whom?
Source: PLoS One. 2024 Aug 28;19(8):e0307220. doi: 10.1371/journal.pone.0307220 (PMC11356444; doi:10.1371/journal.pone.0307220)
Supplement: S1 Appendix — (DOCX) [file pone.0307220.s001.docx]

APPENDIX

Table A1

| Variation from **2018** to 2019 | | P1 | P2 | P3 | P4 | 2019 (%) |
| --- | --- | --- | --- | --- | --- | --- |
| Age | <65 years | +1.55% | +1.67% | +0.49% | +1.45% | 24.0% |
|  | 65 to 74 years | +0.39% | +1.37% | +1.73% | +2.04% | 22.0% |
|  | 75 to 84 years | +0.59% | -0.42% | +0.69% | +0.29% | 26.9% |
|  | >84 years | +2.70% | +2.28% | +2.00% | +1.89% | 26.1% |
| Health Status | No complication | +1.38% | +1.84% | +1.13% | +2.29% | 91.9% |
|  | With Complications | -3.97% | -0.51% | +1.36% | +2.14% | 8.1% |
|  | No consequence | -1.10% | +1.31% | +2.45% | +2.28% | 49.9% |
|  | With consequences | +3.86% | +2.19% | +1.6% | +1.3% | 51.1% |
| Deprivation index | Above Q1 | +3.14% | +2.58% | +2.17% | +0.09% | 19.4% |
|  | Q1-Q2 | +1.30% | +2.34% | +0.63% | +1.89% | 21.86% |
|  | Q2-Q3 | +2.59% | +2.84% | +1.22% | +2.51% | 22.09% |
|  | Q3-Q4 | +0.26% | -0.41% | -0.35% | +1.87% | 20.24% |
|  | Over Q4 | +1.69% | +2.02% | +0.47% | +1.91% | 16.43% |

*Source: PMSI-MCO, INSEE-INSERM, exhaustive administrative database for stroke patients _ 2018-2019*

Note: P _ period, from 1 to 4.

P1: 01/01/2020 to 16/03/2020

P2: 17/03/2020 to 09/05/2020

P3: 10/05/2020 to 15/11/2020

P4: 16/11/2020 to 17/12/2020

For the year 2018, we do not have information on the exact day of admission or discharge. We have the month of discharge. This information is also contained for 2019-database and 2020-database. Besides, we do not have information on chronic disease declared during the hospital’s stay. About the vulnerability, we do not have information to compute this index for the year 2018.

Table A2. Pre-COVID-19 pandemic period: Regression coefficients on variation in inpatients’ healthcare-seeking behavior per 10’000 inhabitants

*Y_ij_*: Reducing change in healthcare access for the subgroup *ij*

|  | Model A | | Model B | | Model C | | Model D | |
| --- | --- | --- | --- | --- | --- | --- | --- | --- |
|  | Coef. | P>t | Coef. | P>t | Coef. | P>t | Coef. | P>t |
|  |  |  |  |  |  |  |  |  |
| Senior  (over 75) | 3.278 | 0.576 | 3.842 | 0.283 | 5.023 | 0.103 | 2.498 | 0.248 |
| With Complications | -2.958 | 0.328 | -3.706 | 0.128 | -4.043 | 0.148 | -1.824 | 0.431 |
| With Consequences | -4.514 | 0.442 | -0.7141 | 0.862 | -0.687 | 0.842 | -0.450 | 0.807 |
| Rural areas | 0.505 | 0.836 | 1.091 | 0.760 | 0.944 | 0.768 | 1.059 | 0.509 |
| Vulnerability index | -5.618* | 0.086 | -7.000* | 0.317 | -5.280* | 0.065 | -4.211* | 0.072 |
| Deprivation : Better-off (Q1) | 3.191 | 0.194 |  |  |  |  |  |  |
| Deprivation : the deprived (Q4 & Q5) |  |  | -1.982 | 0.719 |  |  |  |  |
| Deprivation : the most deprived (Q5) |  |  |  |  | -3.508 | 0.179 | -2.904 | 0.170 |
| Chronic diseases | |  |  |  |  |  | -1.637 | 0.106 |
| Constant | 12.437*** | 0.000 | 16.953*** | 0.001 | 17.365*** | 0.000 | 9.118*** | 0.000 |
|  |  |  |  |  |  |  |  |  |
| R-squared (%) | 43.53 | | 30.31 | | 32.13 | | 32.25 | |

*Source: PMSI-MCO, INSEE-IRCOM, INSEE-INSERM, exhaustive administrative database for stroke patients _ 2019-2020*

Note: During the pre COVID-19 pandemic period P_1_ _ 01/01/2020 to 16/03/2020

*: p<.1; **: p<.05; ***: p<.01

Table A3. During the lockdown period: Regression coefficients on variation in inpatients’ healthcare-seeking behavior per 10’000 inhabitants

*Y_ij_*: Reducing change in healthcare access for the subgroup *ij*

|  | Model A | | | | Model B | | | Model C | | Model D | | | | Model E | | |
| --- | --- | --- | --- | --- | --- | --- | --- | --- | --- | --- | --- | --- | --- | --- | --- | --- |
|  | Coef. | P>t | | | Coef. | P>t | | Coef. | P>t | Coef. | P>t | | | Coef. | P>t | |
|  |  |  | | |  |  | |  |  |  |  | | |  |  | |
| Senior  (over 75) | 8.738 | | 0.379 | 8.027 | | 0.299 | 8.501 | | 0.361 | 3.944 | | 0.303 | 2.188 | | | 0.244 |
| With Complications | -8.412*** | | 0.000 | -8.947*** | | 0.000 | -6.216*** | | 0.000 | -6.600*** | | 0.000 | -6.916*** | | | 0.000 |
| With Consequences | -4.103** | | 0.026 | -4.514** | | 0.047 | -2.006 | | 0.111 | -2.015 | | 0.109 | -1.564* | | | 0.080 |
| Rural areas | 17.366 | | 0.383 | 15.997 | | 0.341 | 16.923 | | 0.372 | 9.195 | | 0.317 | 4.605 | | | 0.214 |
| Vulnerability index | -26.330*** | | 0.000 | -25.125*** | | 0.000 | -25.887*** | | 0.000 | -24.661*** | | 0.000 | -22.872*** | | | 0.000 |
| Q1 (quintile of deprivation index) | -2.033 | | 0.202 |  | |  |  | |  |  | |  |  | | |  |
| Q4 & Q5 (quintiles of deprivation index) |  | |  | -1.364 | | 0.143 |  | |  |  | |  |  | | |  |
| Q5 (quintile of deprivation index) |  | |  |  | |  | +4.442 | | 0.210 | +3.590 | | 0.321 | +2.032 | | | 0.450 |
| Chronic diseases |  | |  |  | |  |  | |  | 8.305 | | 0.031 | 4.5293 | | | 0.016 |
| Regional crisis |  | |  |  | |  |  | |  |  | |  | 7.125*** | | | 0.000 |
| Constant | 93.232*** | | 0.000 | 82.241*** | | 0.000 | 88.862*** | | 0.000 | 82.284*** | | 0.000 | 74.997*** | | | 0.000 |
|  |  | |  |  | |  |  | |  |  |  | |  | | |  |
| R-squared (%) | 14.97 | | | | 18.16 | | | 21.31 | | 24.97 | | | | 26.55 | | |

*Source: PMSI-MCO, INSEE-IRCOM, INSEE-INSERM, exhaustive administrative database for stroke patients _ 2019-2020*

Note: During the lockdown period P_2_ _ 17/03/2020 to 09/05/2020

*: p<.1; **: p<.05; ***: p<.01

Table A4. “In between” period: Regression coefficients on variation in inpatients’ healthcare-seeking behavior per 10’000 inhabitants

*Y_ij_*: Reducing change in healthcare access for the subgroup *ij*

|  | Model A | | Model B | | | Model C | | | Model D | | |  |
| --- | --- | --- | --- | --- | --- | --- | --- | --- | --- | --- | --- | --- |
|  | Coef. | P>t | | Coef. | P>t | | Coef. | P>t | | Coef. | P>t | |
|  |  |  | |  |  | |  |  | |  |  | |
| Senior  (over 75) | 5.833** | 0.045 | | 5.085** | 0.043 | | 5.649** | 0.027 | | 3.033*** | 0.006 | |
| With Complications | -6.777* | 0.021 | | -7.283*** | 0.004 | | -6.635** | 0.010 | | -4.019*** | 0.000 | |
| With Consequences | -3.903 | 0.176 | | -4.043 | 0.105 | | -3.789 | 0.134 | | -2.132* | 0.051 | |
| Rural areas | 7.833*** | 0.008 | | 8.184*** | 0.002 | | 7.621*** | 0.003 | | 4.675*** | 0.000 | |
| Vulnerability index | -2.678 | 0.351 | | -1.859 | 0.452 | | -2.493 | 0.321 | | -1.674 | 0.125 | |
| Q1 (quintile of deprivation index) | -2.368 | 0.409 | |  |  | |  |  | |  |  | |
| Q4 & Q5 (quintiles of deprivation index) |  |  | | 3.508* | 0.065 | |  |  | |  |  | |
| Q5 (quintile of deprivation index) |  |  | |  |  | | 4.522* | 0.071 | | 2.364** | 0.031 | |
| Chronic diseases | |  | |  |  | |  |  | | 0.981 | 0.367 | |
| Constant | 9.023 | 0.019 | | 10.861 | 0.001 | | 9.473 | 0.006 | | 5.805 | 0.000 | |

| R-squared (%) | 15.85 | 25.11 | 24.91 | 26.81 |
| --- | --- | --- | --- | --- |

*Source: PMSI-MCO, INSEE-IRCOM, INSEE-INSERM, exhaustive administrative database for stroke patients _ 2019-2020*

Note: During the “in between” period P_3_ _ 10/05/2020 to 15/11/2020

Table A5. During the shutdown period: Regression coefficients on variation in inpatients’ healthcare-seeking behavior per 10’000 inhabitants

*Y_ij_*: Reducing change in healthcare access for the subgroup *ij*

|  | Model A | | | | Model B | | | Model C | | Model D | | | Model E | | |
| --- | --- | --- | --- | --- | --- | --- | --- | --- | --- | --- | --- | --- | --- | --- | --- |
|  | Coef. | P>t | | | Coef. | P>t | | Coef. | P>t | Coef. | P>t | | Coef. | P>t | |
|  |  |  | | |  |  | |  |  |  |  | |  |  | |
| Senior  (over 75) | 10.177* | | 0.066 | 9.546* | | 0.068 | 9.837** | | 0.043 | 2.960*** | 0.005 | 3.112*** | | | 0.002 |
| With Complications | -16.274*** | | 0.004 | -15.764*** | | 0.003 | -14.855*** | | 0.003 | -4.973*** | 0.000 | -5.585*** | | | 0.000 |
| With Consequences | -4.514 | | 0.409 | -4.964 | | 0.337 | -5.037 | | 0.294 | -0.483 | 0.645 | -0.134 | | | 0.893 |
| Rural areas | -3.874 | | 0.479 | -3.436 | | 0.505 | -3.109 | | 0.516 | -0.968 | 0.353 | -1.054 | | | 0.288 |
| Vulnerability index | -6.674* | | 0.073 | -5.982* | | 0.083 | -6.455 | | 0.101 | -2.621** | 0.046 | -2.128* | | | 0.059 |
| Q1 (quintile of deprivation index) | -9.655* | | 0.081 |  | |  |  | |  |  |  |  | | |  |
| Q4 & Q5 (quintiles of deprivation index) |  | |  | -1.982 | | 0.700 |  | |  |  |  |  | | |  |
| Q5 (quintile of deprivation index) |  | |  |  | |  | 6.746* | | 0.061 | 3.788*** | 0.008 | 3.046*** | | | 0.002 |
| Chronic diseases |  | |  |  | |  |  | |  | 1.310 | 0.209 | 1.285 | | | 0.195 |
| Regional crisis |  | |  |  | |  |  | |  |  |  | 4.935*** | | | 0.000 |
| Constant | 29.379 | | 0.000 | 26.246 | | 0.000 | 27.701 | | 0.000 | 8.116 | 0.000 | 10.723 | | | 0.000 |
|  |  | |  |  | |  |  | |  |  |  |  | | |  |
| R-squared (%) | 14.97 | | | | 18.16 | | | 21.31 | | 24.97 | | | 26.55 | | |

*Source: PMSI-MCO, INSEE-IRCOM, INSEE-INSERM, exhaustive administrative database for stroke patients _ 2019-2020*

Note: During the “in between” period P_4_ _ 16/11/2020 to 17/12/2020
