# Supplementary material for: Stroke but no hospital admission: Lost opportunity for whom?
Source: PLoS One. 2024 Aug 28;19(8):e0307220. doi: 10.1371/journal.pone.0307220 (PMC11356444; doi:10.1371/journal.pone.0307220)
Supplement: Attachment — Submitted filename: Response to ReviewersV2.docx [file pone.0307220.s002.docx]

Reviewer #1

Thanks for this article. Here are my comments, highlighting specific points in your manuscript.

Dear reviewer, thank you for your feedback on our manuscript. We have carefully considered all your suggestions and made the necessary revisions. We hope that you find the revised version satisfactory.

***
Computed variables: “From the patient’s postcode, we set up a dummy for residences in a rural area.”
Could you explain why you chose to include this very specific variable even though you also include a variable about the deprivation index?

We tested several models and found that the main results presented in the paper remain unchanged. The models we tested included:

- A model that considered deprivation index variables
- A model that considered a dummy for residences in a rural area
- A model that considered both deprivation index variables and a dummy for residences in a rural area

However, we have decided to present the model that considers both types of information. This is because living in a rural area does not necessarily mean that the area is poor, but it does mean that there is less access to healthcare. By presenting the model with both types of variables, readers can be confident that the results obtained are not due to the absence of one of these two variables.

We have included a comment in the text.

*For each model, we consider a variable for rural residences and a deprivation index. It is important to note that living in a rural area does not necessarily mean the area is impoverished. However, regardless of the area's level of wealth, living in a rural area often means less access to healthcare.*

***
Computed variables: “A dummy for enrollment in universal health protection, called PUMa, is defined. This insurance (Protection Universelle Maladie) is part of public health insurance. It is designed for people outside the labor market and no longer seeking employment.”
Could you also explain why you chose to include this variable even though you included a variable about the deprivation index and vulnerability?

It should be noted that not all individuals who are in economic or social vulnerability situations are covered by a specific insurance scheme designed for the most disadvantaged. One of the main reasons for this is that eligibility does not necessarily mean that the individual will take out this insurance, which is known as PUMa. For this reason, we have made a distinction between being in a situation of significant vulnerability and being covered by this insurance.

In the text, we have added the following sentence:

*It is important to understand that not all people who are eligible for PUMa programs actually use the program for various reasons, such as difficulty in asserting their right to the program. Additionally, individuals who are identified as vulnerable by healthcare teams may not be eligible for this program. However, it is worth noting that the program offers universal access to healthcare.*

***
Computed variables: “The extreme vulnerability dummy variable is defined as having at least one of these individual characteristics”. I do not understand why you decide not disentangling between people with one characteristic and people two or more characteristic.
Could you at least present the distribution among the people you consider in your analysis?

We agree that we may do different assumptions on the population defined as extreme vulnerability. However, the greater the refinement of population characteristics, the smaller the cell sizes. Therefore there is a trade-off between statistical power and the level of detail of the variables considered.

As you suggested, we present here the distribution among the population of this study. Table 1 present in last column, the percent of extreme vulnerable population in the stroke population in 2019. There is 7.5% of the population defined as in extreme vulnerable situation.

In the text, we have added the following sentence:

*Table 1, last column, presents the percentage of the stroke population in extreme vulnerable* *situations, which was 7.5% in 2019.*

***
Specific computed variables for the Covid-19 pandemic: “In France, the COVID-19 pandemic can be divided into four periods over 2020 (P1 to P4), according to intensity.”
Why did you choose to use the four periods calculated based on government measures rather than considering the evolution of the pandemic? People in France were aware of all the figures, presented each day by the government, and, likely, they did not adjust their behavior based on the progression of waves as much as the evolution of social measures. Could you discuss this point?

Dans la section « discussion » nous avons donc ajouté le paragraphe suivant :

We agree that the decision to focus on government measures to define the peaks of the pandemic needs to be discussed. We have tried to take periods that could be objectivized. They are debatable, but these periods have the advantage of being well-documented and of having well-established start and end points. As a result, we found them less debatable than other periods based on the social behavior of individuals. The health crisis was marked by different phases of acceptance for different groups in society. These groups depend on social, economic, environmental, religious and cultural criteria. It is therefore difficult to reach a consensus on the periods to be taken into account based on these groups. Moreover, the geographical differences of the health crisis add to the difficulty of reaching a consensus on these groups for the French population as a whole.

However, we fully agree that this choice needs to be discussed. Another choice could have been just as relevant, depending on the perspective adopted. For example, focusing on cultural aspects would have led us to take other periods into account.

We have therefore added the following paragraph to the "discussion" Section:

*The study based the calculation of four periods on government measures instead of the progression of the pandemic. Although the French population had access to daily figures presented by the government, there is a possibility that some groups did not modify their behavior based on the advancement of the pandemic, but rather on the changing social measures.*

***

Specific computed variables for the COVID-19 pandemic: “We created a binary variable by period (regional crisis) equal to one when the patient’s residence was in a critical situation area.”
Could you precisely explain the indicator you have used to classify the French regions? Additionally, this classification is based on epidemic indicators, which is different from your definitions of the sub-periods.

We have used what the regulator has defined as risk regions in the first two waves. This is an administrative definition. It is based on epidemiological indicators, but it is true that it is not a definition based on epidemiological indicators.

| French Regions/States | |
| --- | --- |
| P2 | P4 |
| Grand-est | Auvergne Rhone Alpes |
| Ile de France | Ile de France |
|  | PACA |
|  | Hauts de France |
|  | Occitanie |

In preliminary work, we used the departments and the evolution of the Covid hospitalization rate per 1,000 inhabitants. The main ones presented here were also obtained.

|  |  | Covid hospitalization rate per  1,000 inhabitants | |
| --- | --- | --- | --- |
| French departement | | P2 | P4 |
| **1** | Ain | 0,8 | 2,2 |
| **2** | Aisne | 2,0 | 2,4 |
| **3** | Allier | 0,7 | 3,2 |
| **4** | Alpes-de-Haute-Provence | 0,6 | 2,5 |
| **5** | Hautes-Alpes | 1,4 | 4,5 |
| **6** | Alpes-Maritimes | 0,9 | 2,0 |
| **7** | Ardèche | 1,4 | 3,1 |
| **8** | Ardennes | 0,9 | 2,0 |
| **9** | Ariège | 0,3 | 1,0 |
| **10** | Aube | 2,4 | 2,1 |
| **11** | Aude | 0,9 | 1,1 |
| **12** | Aveyron | 0,6 | 1,4 |
| **13** | Bouches-du-Rhône | 2,0 | 3,0 |
| **14** | Calvados | 0,5 | 1,6 |
| **15** | Cantal | 0,7 | 2,2 |
| **16** | Charente | 0,2 | 0,6 |
| **17** | Charente-Maritime | 0,4 | 0,6 |
| **18** | Cher | 1,0 | 2,6 |
| **19** | Corrèze | 0,9 | 1,2 |
| **2A** | Corse-du-Sud | 0,4 | 0,9 |
| **2B** | Haute-Corse | 0,5 | 0,7 |
| **21** | Côte-d'Or | 1,8 | 2,9 |
| **22** | Côtes d'Armor | 0,5 | 0,6 |
| **23** | Creuse | 0,7 | 1,8 |
| **24** | Dordogne | 0,2 | 0,7 |
| **25** | Doubs | 1,8 | 2,2 |
| **26** | Drôme | 1,5 | 2,7 |
| **27** | Eure | 0,6 | 1,3 |
| **28** | Eure-et-Loir | 1,8 | 2,0 |
| **29** | Finistère | 0,3 | 0,5 |
| **30** | Gard | 0,6 | 1,9 |
| **31** | Haute-Garonne | 0,7 | 1,4 |
| **32** | Gers | 0,6 | 0,8 |
| **33** | Gironde | 0,8 | 1,0 |
| **34** | Hérault | 0,7 | 1,6 |
| **35** | Ille-et-Vilaine | 0,5 | 1,0 |
| **36** | Indre | 1,3 | 1,5 |
| **37** | Indre-et-Loire | 0,9 | 1,2 |
| **38** | Isère | 0,7 | 3,4 |
| **39** | Jura | 1,4 | 3,5 |
| **40** | Landes | 0,3 | 1,1 |
| **41** | Loir-et-Cher | 1,1 | 1,4 |
| **42** | Loire | 1,9 | 3,9 |
| **43** | Haute-Loire | 0,3 | 3,4 |
| **44** | Loire-Atlantique | 0,6 | 0,9 |
| **45** | Loiret | 1,3 | 1,3 |
| **46** | Lot | 0,6 | 0,8 |
| **47** | Lot-et-Garonne | 0,3 | 1,2 |
| **48** | Lozère | 0,3 | 2,9 |
| **49** | Maine-et-Loire | 1,0 | 1,6 |
| **50** | Manche | 0,5 | 1,0 |
| **51** | Marne | 2,2 | 1,9 |
| **52** | Haute-Marne | 2,2 | 2,6 |
| **53** | Mayenne | 0,7 | 1,7 |
| **54** | Meurthe-et-Moselle | 2,4 | 2,4 |
| **55** | Meuse | 3,0 | 2,5 |
| **56** | Morbihan | 0,7 | 0,8 |
| **57** | Moselle | 3,5 | 2,3 |
| **58** | Nièvre | 0,6 | 1,7 |
| **59** | Nord | 1,3 | 2,7 |
| **60** | Oise | 2,1 | 1,4 |
| **61** | Orne | 1,0 | 2,3 |
| **62** | Pas-de-Calais | 1,2 | 2,1 |
| **63** | Puy-de-Dôme | 0,4 | 2,1 |
| **64** | Pyrénées-Atlantiques | 0,4 | 1,6 |
| **65** | Hautes-Pyrénées | 0,7 | 2,0 |
| **66** | Pyrénées-Orientales | 0,9 | 0,9 |
| **67** | Bas-Rhin | 3,3 | 1,9 |
| **68** | Haut-Rhin | 4,7 | 2,0 |
| **69** | Rhône | 2,2 | 3,7 |
| **70** | Haute-Saône | 1,5 | 2,1 |
| **71** | Saône-et-Loire | 1,7 | 4,3 |
| **72** | Sarthe | 0,9 | 1,7 |
| **73** | Savoie | 1,0 | 4,4 |
| **74** | Haute-Savoie | 1,5 | 3,4 |
| **75** | Paris | 4,6 | 2,3 |
| **76** | Seine-Maritime | 0,9 | 1,7 |
| **77** | Seine-et-Marne | 2,6 | 1,6 |
| **78** | Yvelines | 2,1 | 1,5 |
| **79** | Deux-Sèvres | 0,3 | 1,0 |
| **80** | Somme | 1,6 | 1,5 |
| **81** | Tarn | 0,5 | 1,7 |
| **82** | Tarn-et-Garonne | 0,3 | 1,7 |
| **83** | Var | 1,1 | 1,8 |
| **84** | Vaucluse | 0,5 | 3,3 |
| **85** | Vandée | 0,4 | 0,8 |
| **86** | Vienne | 0,5 | 1,1 |
| **87** | Haute-Vienne | 0,5 | 1,3 |
| **88** | Vosges | 2,7 | 3,3 |
| **89** | Yonne | 1,3 | 2,6 |
| **90** | Territoire de Belfort | 5,5 | 6,6 |
| **91** | Essonne | 2,5 | 1,7 |
| **92** | Hauts-de-Seine | 3,9 | 2,1 |
| **93** | Seine-Saint-Denis | 3,1 | 1,9 |
| **94** | Val-de-Marne | 4,2 | 2,4 |
| **95** | Val-D'Oise | 2,9 | 1,8 |
| **971** | Guadeloupe | 0,3 | 0,7 |
| **972** | Martinique | 0,4 | 0,7 |
| **973** | Guyane | 0,2 | 0,7 |
| **974** | La Réunion | 0,2 | 0,5 |
| **976** | Mayotte | 1,0 | 0,4 |

We have opted to present the results with the "regional crisis" variable, as we feel the results are more readable for a reader unfamiliar with France. But above all, we wanted to maintain consistency between the political decisions to determine peak crisis periods and the political decisions to define regions in health crisis from the regulator's point of view.

We have therefore added the following paragraphs:

- Section 2.3, we added

*The detailed information is given Table A6 in Appendix.*

[…]

In the Appendix, we added the Table A6

*Table A6 : Regional crisis description*

| **French Regions/States** | |
| --- | --- |
| **P2** | **P4** |
| Grand-est | Auvergne Rhone Alpes |
| Ile de France | Ile de France |
|  | PACA |
|  | Hauts de France |
|  | Occitanie |

- "discussion" Section, we added,

*We have chosen to display the outcomes with the "regional crisis" factor as we aimed to ensure consistency between the political decisions that identify peak crisis periods and the political decisions that define regions in a health crisis, from the regulator's perspective. However, in preliminary work, we used Covid hospitalization rate per 1,000 inhabitants at the department level. The main results presented in this paper were also obtained.*

***
Empirical framework: “This paper assumes that, without the COVID-19 pandemic, stroke patient distribution in 2020 should have followed stroke patient distribution in 2019. As a comparative analysis, we checked that this is observed when comparing 2018 to 2019. The method described below has been used in the years 2018-2019. The differences in magnitudes of in-admission rates from 2018 to 2019 are negligible. These differences are used as a benchmark to judge the 2019-2020 differences. “
Could you enlarge your time window to assess the validity of this argument; maybe use data before 2018? 2019 was a very specific year for the evolution of life expectancy in France, and even if it is not visible for strokes, I would appreciate it if you could discuss this point.

We could have extended our time windows to assess the validity of this argument. It's possible that the two years prior to COVID were particular years for stroke compared with earlier years. However, we are unable to do so today, as access to the raw data is no longer available. We do have access to the database built to produce the results presented here. However, we no longer have access to the raw data. The constructed databases are also accessible for a limited time, as they remain sensitive data within the meaning of the RGPD/CNIL.

We have added this remark in the "discussion" section of the paper

*In this paper, we used 2018 and 2019 data as a benchmark. To enhance model robustness, it would have been interesting to expand the time frame and evaluate the sensitivity of the outcomes.*

***
Empirical framework: “The construct is the following: we set up groups of patients i according to a set of variables.“
Could you be more precise about the construction of all the groups? You should introduce in this paragraph the explanations you give in the results sections.

As recommended, we have relocated the explanatory paragraph from the "results" section to section 3.1 where it follows the sentence quoted by the reviewer.

In Section 3.1, we now can read the following paragraph

*Each subgroupij is defined by the period Pj crossed year. In the regression results presented here, the subgroups of patients are computed according to the following dummy variables: over 74 years old, with complications, with consequences, with vulnerability, rural areas, chronic diseases, quintile of the French deprivation index (Fdep) for the most deprived (Q5), for the deprived (Q4), for the better-off (Q1), and region with local COVID-19 pandemic. There are 28 =256 groupsi. They are subdivided into four according to the period Pj. Therefore, there are 1’024 subgroupsij. As sensitivity analysis, we run models with alternative sets of variables to set up groups of patients. Results are available upon request.*

In Section 4.3, we now can read the following paragraph

*As previously presented, the dependent variable Yij is the variation in healthcare access. The greater the variation is, the more important the healthcare renouncement is. As explained in Section 3.1 “A non-parametric model”, the subgroupij is determined by specific clinical, demographic, vulnerability, and deprivation factors setting up the group i for a given period j.*

***
Results: “As sensitivity analysis, we run models with alternative sets of variables to set up groups of patients. Results are available upon request.“
Could you be more precise about the construction of these alternative groups, and maybe present the results in the appendix?

We analyzed different age ranges to maintain statistical consistency with four age groups. Additionally, we combined health status with geographical area, while excluding deprivation and vulnerability variables to ensure that the number of cells was statistically sufficient. We also considered different levels of vulnerability without taking into account the deprivation index for the same reason. We examined the last deciles and quartiles for the deprivation index and constructed less detailed groups with one age class (over or under 80) and one health status class (with a health problem) to obtain robust cell sizes and study the sensitivity of the results.

All these changes enabled us to validate the robustness of our results.

***
Discussion: “It suggests that after an exogenous shock affecting the whole population, the upturn in behaviour to levels seen previous to the COVID-19 pandemic period depends on deprivation factors and age threshold. I am quite sure that there exists a large literature about this question.“
Could you cite this literature in the discussion? Less specifically, I am a little be surprised that no article is quoted in the discussion part.

We agree with the reviewer. And I added a paragraph in the discussion Section with the related literature.

*These results are in line with those of Teo et al. (2020), Gale et al. (2020), Kiss  et al. (2020) and Janke et al. (2022). During the early containment phase of COVID-19 at Hong Kong, Teo et al. show a prolongation in stroke onset to hospital arrival time and a significant reduction in individuals arriving at the hospital within 4.5 hours and presenting with transient ischemic attack. Leira et al. (2020) explained that the coronavirus 2019 (COVID-19) pandemic required drastic changes in allocation of resources, which affected the delivery of stroke care. As well, Montaner et al. (2020) showed that COVID-19 pandemic was disruptive for acute stroke pathways.*

We added these following references :

Gale R, Eberlein S, Fuller G, Khalil C, Almario CV, Spiegel BMR. Public perspectives on decisions about emergency care seeking for care unrelated to COVID-19 during the COVID-19 pandemic. JAMA Network Open. 2021;4:e2120940.

Janke AT, Gettel CJ, Haimovich A, Kocher KE, Krumholz HM, Venkatesh AK. Changes in emergency department arrival times for acute myocardial infarction during the COVID-19 pandemic suggest delays in care seeking. Circulation. 2022;15(2):e008402. doi:10.1161/CIRCOUTCOMES.121.008402

Kiss P, Carcel C, Hockham C, Peters SAE. The impact of the COVID-19 pandemic on the care and management of patients with acute cardiovascular disease: a systematic review. Eur Heart J Qual Care Clin Outcomes. 2021;7(1):18–27. doi:10.1093/ehjqcco/qcaa084

Leira EC, Russman AN, Biller J, Brown DL, Bushnell CD, Caso V, Chamorro A, Creutzfeldt CJ, Cruz-Flores S, Elkind MSV, Fayad P, Froehler MT, Goldstein LB, Gonzales NR, Kaskie B, Khatri P, Livesay S, Liebeskind DS, Majersik JJ, Moheet AM, Romano JG, Sanossian N, Sansing LH, Silver B, Simpkins AN, Smith W, Tirschwell DL, Wang DZ, Yavagal DR, Worrall BB. Preserving stroke care during the COVID-19 pandemic: Potential issues and solutions. Neurology. 2020 Jul 21;95(3):124-133. doi: 10.1212/WNL.0000000000009713. Epub 2020 May 8. PMID: 32385186; PMCID: PMC7455350.

Montaner J., Barragán-Prieto A., Pérez-Sánchez S., Escudero-Martínez I., Moniche F., Sánchez-Miura J., Ruiz-Bayo L., González A. [Break in the Stroke Chain of Survival due to COVID-19](https://www.ahajournals.org/doi/abs/10.1161/STROKEAHA.120.030106). Stroke. 2020;51:2307–2314

Teo KC, Leung WCY, Wong YK, Liu RKC, Chan AHY, Choi OMY, Kwok WM, Leung KK, Tse MY, Cheung RTF, Tsang AC, Lau KK. Delays in Stroke Onset to Hospital Arrival Time During COVID-19. Stroke. 2020 Jul;51(7):2228-2231. doi: 10.1161/STROKEAHA.120.030105. Epub 2020 May 20. PMID: 32432998; PMCID: PMC7258759.

***
Tables: In Table 1, I do not understand the definition of the variable in the last column. Moreover, I am sure that you could present these results with a figure and not in a table.This should help the readers to easily grasp the magnitude of your results.

Table 1's last column has been renamed to clarify that it refers to the distribution of the variables.

Reviewer #2:

Dear reviewer, thank you for your feedback on our manuscript. We have carefully considered all your suggestions and made the necessary revisions. We hope that you find the revised version satisfactory.

> As regards the content and the statistical analysis (point 2. above):

It is not clear if a panel methodology was used. Colinearity of the explanatory variables was not checked. Post-tests on residuals were not run. If panel estimation was carried out, the Hausman test was not run.

As suggested by the Reviewer #1, we added information in the 3.1 section, « A non-parametric method »

*“We set up groups of patients i according to a set of variables. Then, for each group i defined, we considered four periods corresponding to the time ranges of the COVID-19 pandemic phases. These subgroups_ij_ are computed for the year 2019 (2019-subgroup_ij_) and for the year 2020 (2020- subgroup_ij_).*

*Each subgroup_ij_ is defined by the period P_j_ crossed year. In the regression results presented here, the subgroups of patients are computed according to the following dummy variables: over 74 years old, with complications, with consequences, with vulnerability, rural areas, chronic diseases, quintile of the French deprivation index (Fdep) for the most deprived (Q5), for the deprived (Q4), for the better-off (Q1), and region with local COVID-19 pandemic. There are 2^8^ =256 groups_i_. They are subdivided into four according to the period P_j_. Therefore, there are 1’024 subgroups_ij_. As sensitivity analysis, we run models with alternative sets of variables to set up groups of patients. Results are available upon request.*

*Next, for each subgroup_ij_, we computed the variation (%) in the number of inpatients over the two years (2019 and 2020). The variation is computed with the year 2019 as a reference. Using variation allows us to eliminate the magnitude of the difference in number of inpatients between groups. We introduce weightings in the model to distinguish strong variations exclusively for marginal groups of patients in the distribution. This variation_ij_ is then weighted by the size of the 2019-subgroup_ij_ in the distribution. As sensitivity tests for results, we selected different sets of variables for group compositions.*

*For this method, a limitation is that we cannot get standard deviations. Indeed, for each subgroup_ij_ , we compute a unique sum. Therefore, the only way to evaluate the validity of results is by comparing these figures with figures obtained for the years 2018 to 2019. We do not have any statistical tests to assess the impact of deprivation and vulnerability factors on healthcare access behavior. We then use a parametric weighted least squares model.”*

Besides, we can also add that we begin by using panel data and transform it to measure the difference between two years - the reference year and the year impacted by the health crisis. We then analyze this new database of variations between the two years, which is no longer a panel database.

By presenting models A to E, we can determine the correlation between the variables. Here, the variables that are present in the model are those whose coefficients we wish to have. However, we agree that the level of correlation between them may prevent us from correctly interpreting these coefficients. Presenting models A to E allows us to observe the evolution of the coefficients and thus see how the addition of variables affects them.

> As regards the form of the article (Point 4. above), some sentences may be clarified for better understanding, such as:

- Page 4: In « In this paper, using a very innovative method », it woud be useful to precise of which innovative method the authors are referring to, from the start of the paper. In section « 3.1 A non-parametric method » more details could be added to present the model and the methods used, if the character count makes it possible.

See response to the first comment. We changed the section « 3.1 A non-parametric method » and we added more details to present the model and the methods used.

- Page 7: In section « 3.1 A non-parametric method », in « We used a non-parametric method », the authors could precise to which method they are referring to.

We begin by using panel data and transform it to measure the difference between two years - the reference year and the year impacted by the health crisis. We then analyze this new database of variations between the two years, which is no longer a panel database.

- Page 7: In « The subgroupij is determined by specific clinical, demographic, vulnerability, and deprivation factors setting up the group i for a given period j », is it possible to mention, how the subgroups are constituted, with which thresholds for example ?

In Section 3.1, we now can read the following paragraph

*Each subgroupij is defined by the period Pj crossed year. In the regression results presented here, the subgroups of patients are computed according to the following dummy variables: over 74 years old, with complications, with consequences, with vulnerability, rural areas, chronic diseases, quintile of the French deprivation index (Fdep) for the most deprived (Q5), for the deprived (Q4), for the better-off (Q1), and region with local COVID-19 pandemic. There are 28 =256 groupsi. They are subdivided into four according to the period Pj. Therefore, there are 1’024 subgroupsij. As sensitivity analysis, we run models with alternative sets of variables to set up groups of patients. Results are available upon request.*

- Page 8: In « We introduce weightings in the model », the authors may briefly detail which kind of weightings is used or how they are built.

Individuals at risk of having a stroke during a given period in 2019, is defined as the weights.

*In Section 3.2 “ A weighted ordinary least square, the paragraph is the following*

we study the characteristics of the groups of individuals with non-admitted stroke who could have been admitted before the COVID-19 pandemic. The method is based not on individual-level information but on aggregated data to obtain information on the number of individuals at risk in each subgroup*_ij_*. We use weighted least squares to estimate differences between years and periods. The subgroup size, i.e., individuals at risk of having a stroke during a given period in 2019, is defined as the weights.
